# Chemistry of Spontaneous Alkylation of Methimazole with 1,2-Dichloroethane

**DOI:** 10.3390/molecules26227032

**Published:** 2021-11-21

**Authors:** Leo Štefan, Ana Čikoš, Robert Vianello, Ivica Đilović, Dubravka Matković-Čalogović, Miljenko Dumić

**Affiliations:** 1 JGL d.d. Jadran Galenski Laboratorij, 51 000 Rijeka, Croatia; 2 NMR Centre, Ruđer Bošković Institute, 10 000 Zagreb, Croatia; ana.cikos@irb.hr; 3 Laboratory for the Computational Design and Synthesis of Functional Materials, Division of Organic Chemistry and Biochemistry, Ruđer Bošković Institute, 10 000 Zagreb, Croatia; robert.vianello@irb.hr; 4 Department of Chemistry, Faculty of Science, University of Zagreb, 10 000 Zagreb, Croatia; idilovic@chem.pmf.hr (I.Đ.); dubravka@chem.pmf.hr (D.M.-Č.); 5 Department of Biotechnology, University of Rijeka, 51 000 Rijeka, Croatia; mdumic@biotech.uniri.hr

**Keywords:** 1,2-Bis[(1-methyl-1*H*-imidazole-2-yl)thio]ethane, methimazole, 7-methyl-2*H*,3*H*,7*H*-imidazo[2,1-*b*]thiazol-4-ium salts, thiiranium ion, single-crystal X-ray study, isomerization kinetics, time-dependent NMR spectroscopy, computational methods, bis-{2-[(chloroethyl)thio]-1-methyl-1*H*-imidazole}-silver(I)tetrafluoroborate, 2,3-dihydro-3-methyl-1-[(1-methyl-1*H*-imidazole-2-yl)thioethyl]-1*H*-imidazole-2-thione

## Abstract

Spontaneous *S*-alkylation of methimazole (**1**) with 1,2-dichloroethane (DCE) into 1,2-bis[(1-methyl-1*H*-imidazole-2-yl)thio]ethane (**2**), that we have described recently, opened the question about its formation pathway(s). Results of the synthetic, NMR spectroscopic, crystallographic and computational studies suggest that, under given conditions, **2** is obtained by direct attack of **1** on the chloroethyl derivative 2-[(chloroethyl)thio]-1-methyl-1*H*-imidazole (**3**), rather than through the isolated stable thiiranium ion isomer, i.e., 7-methyl-2*H*, 3*H*, 7*H*-imidazo[2,1-*b*]thiazol-4-ium chloride (**4a**, orthorhombic, space group *Pnma*), or in analogy with similar reactions, through postulated, but unproven intermediate thiiranium ion **5**. Furthermore, in the reaction with **1**, **4a** prefers isomerization to the *N*-chloroethyl derivative, 1-chloroethyl-2,3-dihydro-3-methyl-1*H*-imidazole-2-thione (**7**), rather than alkylation to **2**, while **7** further reacts with **1** to form 3-methyl-1-[(1-methyl-imidazole-2-yl)thioethyl]-1*H*-imidazole-2-thione (**8**, monoclinic, space group *P* 2_1_*/c*). Additionally, during the isomerization of **3**, the postulated intermediate thiiranium ion **5** was not detected by chromatographic and spectroscopic methods, nor by trapping with AgBF_4_. However, trapping resulted in the formation of the silver complex of compound **3**, i.e., bis-{2-[(chloroethyl)thio]-1-methyl-1*H*-imidazole}-silver(I)tetrafluoroborate (**6**_,_ monoclinic, space group *P* 2_1_*/c*), which cyclized upon heating at 80 °C to 7-methyl-2*H*, 3*H*, 7*H*-imidazo[2,1-*b*]thiazol-4-ium tetrafluoroborate (**4b**, monoclinic, space group *P* 2_1_*/c*). Finally, we observed thermal isomerization of both **2** and 2,3-dihydro-3-methyl-1-[(1-methyl-1*H*-imidazole-2-yl)thioethyl]-1*H*-imidazole-2-thione (**8**), into 1,2-bis(2,3-dihydro-3-methyl-1*H*-imidazole-2-thione-1-yl)ethane (**9**), which confirmed their structures.

## 1. Introduction

Methimazole (thiamazole, 1-methyl-2,3-dihydro-1*H*-imidazole-2-thione (**1**) is a worldwide used thyrostatic drug [1]. Owing to the structural feature of the ambidentate heterocyclic [N-C-S] type anion, it has also found application as a terminal group in the synthesis of noncyclic crown ethers, as well as in various aspects of coordination chemistry [2,3].

**Figure 1:**
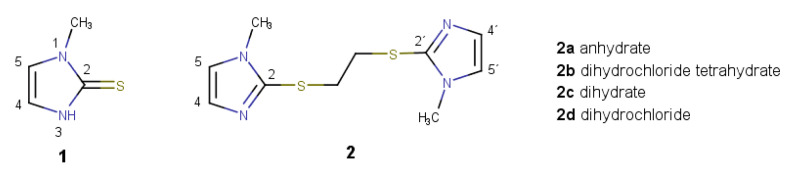
Methimazole (**1**) and 1,2-bis[(1-methyl-1*H*-imidazole-2-yl)thio]ethane (**2**) in different forms (**2a**–**2d**).

In the course of our methimazole preliminary stability study, we recently reported *S*-alkylation of **1** by 1,2-dichloroethane (DCE) in solution [4]. This reaction occurs spontaneously, both in light and dark, at ambient humidity and room temperature within 15 days, leading to the formation of 1,2-bis[(1-methyl-1*H*-imidazole-2-yl)thio]ethane (**2**), primarily isolated in the form of dihydrochloride tetrahydrate (**2b**). Depending on reaction/isolation conditions, the product can be isolated as an anhydrate (**2a**) or dihydrate (**2c**) (Figure 1). Their mutual interconversion was previously described [4].

In the context of the pharmaceutical purity profile, **2** is considered to be very interesting since it is a potential methimazole-related substance, and as such should not be present in the marketed product.

Therefore, aiming to get better insight into the formation pathways leading from **1** to **2**, we carried out synthetic, NMR, crystallographic, and computational studies.

## 2. Results and Discussion

### 2.1. Reaction of Methimazole (**1**) with 1,2-Dichoroethane

The reaction of **1** and DCE, as previously reported, gave 1,2-bis[(1-methyl-1*H*-imidazole-2-yl)thio]ethane dihydrochloride tetrahydrate (**2b**) in the form of colorless plate-shaped crystals. The melting point and the NMR data were identical to the data reported earlier [4]. The remaining mother liquor was analyzed and, after separation, yielded not only the unreacted methimazole, but also the colorless liquid, 2-[(chloroethyl)thio]-1-methyl-*H*-imidazole (**3**, 12.1%). The HRMS and NMR analysis (see Experimental) confirmed the structure of **3**. The reaction of **1** with boiling DCE in the presence of formic acid as a co-solvent gave **3** in a better yield than before (73%), along with **2a**.

**Scheme 1:**
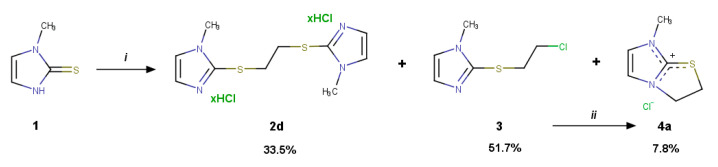
Synthesis of **2d**, **3,** and **4a** from **1** and DCE (dry); (*i*) DCE, reflux, 11 h, (*ii*) r.t, 21 days, 98%.

When the reaction of **1** and dry DCE was performed under reflux without acid (Scheme 1), the product, according to the NMR analysis, contained a mixture of 7-methyl-2*H*, 3*H*, 7*H*-imidazo[2,1*-b*]thiazol-4-ium chloride (**4a**) and bis[(1-methyl-1*H*-imidazole-2-yl)thio]ethane dihydrochloride (**2d**). The latter was isolated by column chromatography in the form of pure colorless crystals. As in the previous case, the mother liquor contained **3,** which was isolated in the form of a colorless oil.

**Figure 2:**
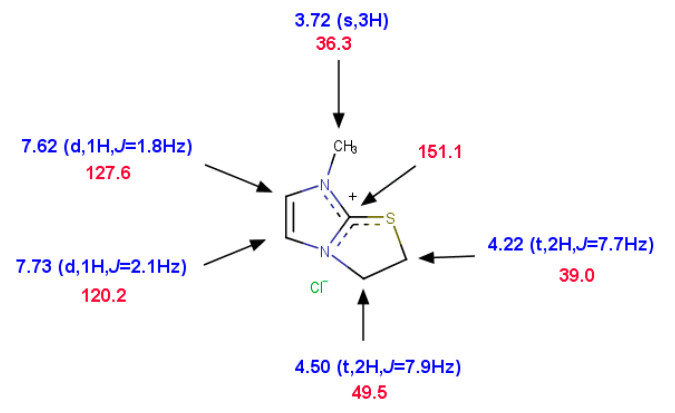
^1^H (600 MHz) (blue), ^13^C (151 MHz) (red) NMR chemical shifts in ppm for **4a** in DMSO-*d_6_* at 25 °C.

**Figure 3:**
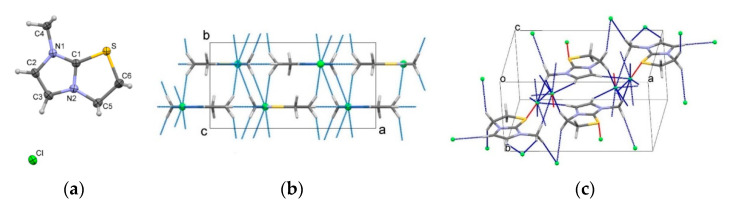
(**a**) Molecular structure of **4a** with the atomic numbering scheme; (**b**) projection of **4a** down the c-axis, (**c**) packing diagram of **4a**. Hydrogen bonds are marked by blue dotted lines and the Cl···S electrostatic interaction by red lines.

The question of **4a** formation was answered when we discovered that the *S*-chloroethyl derivative **3** quantitatively isomerizes into imidazothiazolium chloride **4a** (Scheme 1) after 21 days at room temperature. **4a** was isolated as needle-like crystals, its structure elucidated by NMR spectroscopy (Figure 2) and confirmed by single crystal X-ray diffraction analysis (Figure 3 and Table S1). Indirectly, these results corroborated the structure of the *S*-chloroethyl derivative **3** as well. The imidazothiazolium salt **4a** represents, to the best of our knowledge, the first room temperature stable thiiranium ion isomer reported so far.

Bond lengths, C1-N1 of 1.327(3) Å, C1-N2 of 1.339(3) Å, and S-C1 of 1.725(2) Å in **4a** (Figure 3a) suggest significant delocalization of the electron density and are in correspondence with literature data [5]. In the crystal structure of **4a,** the planar heterobicyclic cations and chloride anions lie in the crystallographic mirror plane. Interatomic distance Cl^−^···S of 3.2125(9) Å is attributed to a short contact suggesting an electrostatic interaction (Figure 3c). There are weak hydrogen bonds of the C-H···Cl type in the range 3.484(2) to 3.7655(12) Å interconnecting the ions into a 3D structure (Figure 3c). 

**Scheme 2:**
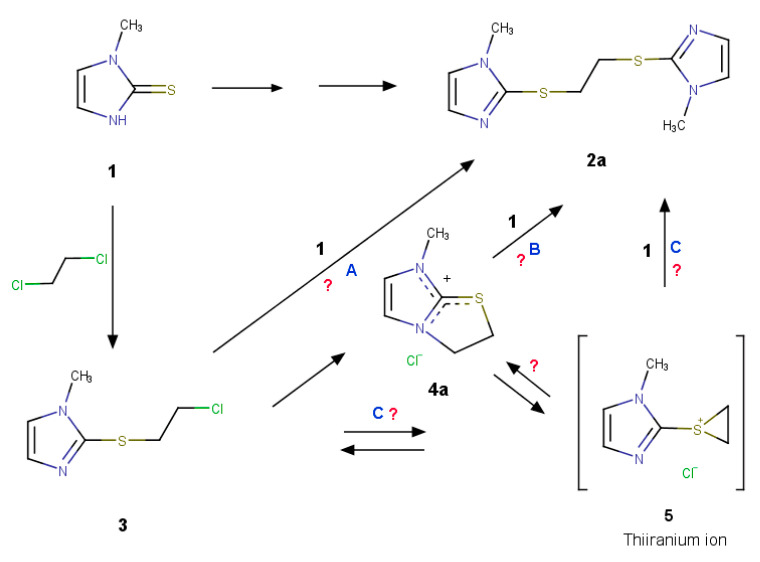
Possible synthetic pathways from **1** to **2** (pathway A—presenting direct attack of **1** at **3**, pathway B—presenting attack of **1** to **4a**, pathway C presenting isomerization of **3** to **5** and **4a** to **5** and attack of **1**).

After isolation and the unambiguous structure determination of **3,** a “sulfur mustard” type compound, and **4a**, formed in the same reaction, we proceeded to elucidate the synthetic pathway to **2.** The possibilities were: direct nucleophilic attack of **1** on the already formed intermediate **3** (pathway A), nucleophilic attack of **1** on the intermediate **4a** (pathway B), or nucleophilic attack of **1** on the thiiranium (episulfonium) ion (**5**)**,** a reactive (fleeting) intermediate in analogy with similar reactions [6,7] (pathway C) (Scheme 2).

Additionally, the highly reactive and, with a few exceptions [8], very unstable thiiranium ions are well described in the literature [6,7,9]. Recently, they were suggested as important synthetic intermediates in regio- and stereoselective sulfenoaminations of thioimidazoles with alkenes [10]. They can also be found as intermediates with a genotoxic potential [10,11] in nucleophilic reactions of “mustard” type compounds similar to our intermediate **3**.

In order to determine which reaction pathway is the most likely, and if the unstable tiiranium ion **5** is present in our reaction, the subsequent research continued both experimentally and theoretically.

### 2.2. Search for Thiiranium Ion Intermediate **5** by NMR Spectroscopy

#### 2.2.1. Kinetics of the S-Chloroethyl Derivative **3** Degradation

To explore if transformation of the *S*-chloroethyl derivative **3** to the stable imidazothiazolium salt **4a** occurs through the thiiranium intermediate **5**, the degradation of **3** in DMSO-*d_6_* at room temperature was monitored by accumulation of time-dependent ^1^H NMR spectra until the reaction completion.

Spontaneous transformation of **3** to **4a** that occurs during the period of 3 months was confirmed, while no signals attributable to the proposed thiiranium derivative **5** were detected in any of the ^1^H spectra. Pilot ^13^C and HSQC spectra also revealed no signals in the region around 40 ppm where Dohn and Casida [12] reported the thiiranium ion formed from cysteine derivatives under superacid conditions. It was concluded that either the mechanism does not involve the thiiranium ion as an intermediate, or, alternatively, it decomposes too quickly to be detected by NMR spectroscopy.

However, after 3 days, an additional set of signals was detected at very low concentration belonging to a so far unknown isomer of **3**, later synthesized, and its structure confirmed as 1-chloroethyl-2,3-dihydro-3-methyl-1*H*-imidazole-2-thione (**7**).

**Figure 4:**
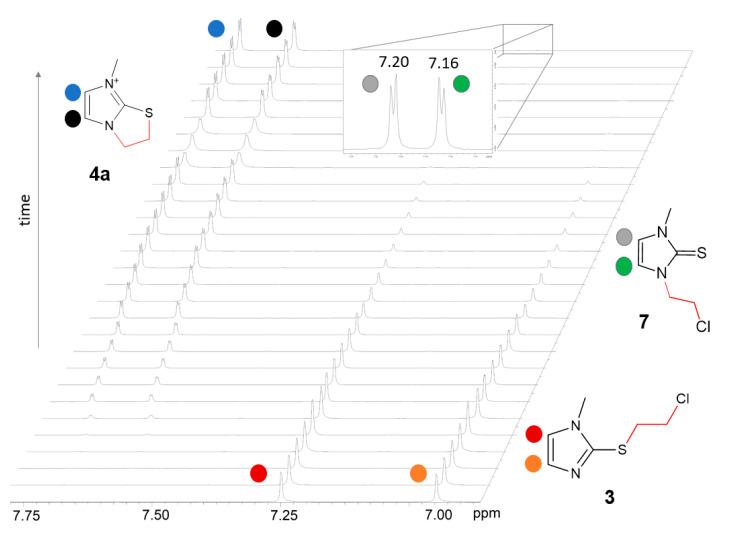
Imidazole region of time-dependent ^1^H NMR spectra in DMSO-*d_6_* for the mixture of **3**, **4a,** and **7**. Imidazole protons of: **3** (presented in orange and red), **7** (presented in green and grey) and **4a** (presented in black and blue).

**Figure 5:**
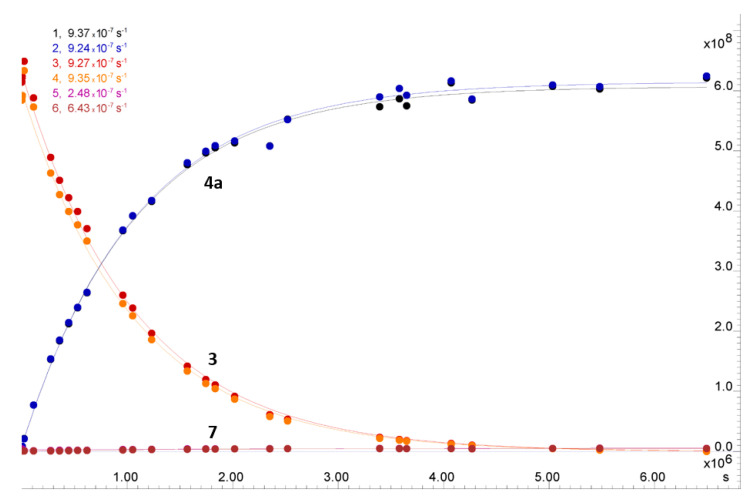
Interconversion kinetics for compounds **3**, **4a,** and **7;** each compound is represented by a fitted curve based on integral values of its two imidazole signals.

**Scheme 3:**
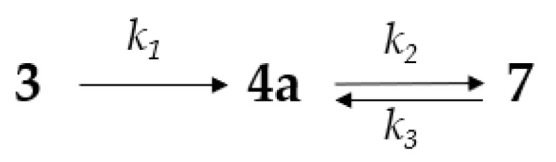
Consecutive degradation of reactant **3** to the two products, **4a** and **7**; *k_1_*,*k_2_*,*k_3_* presents reaction rate constants.

Reaction kinetics of **3** into **4a** and **7** was quantified using integrals of well separated imidazole proton NMR signals for all three species (Figure 4), while the rate constants were calculated with Bruker Dynamics Center V2.6.3 software (Figure 5). Conversion of **3** into compounds **4a** and **7** proceeds to completion after approximately 75 days. The reaction ends in an apparent equilibrium with relative concentrations of 99.1% and 0.94%, for **4a** and **7**, respectively. Therefore, to describe the observed decline in the signal intensity of **3** and the rise in signals belonging to **4a** and **7,** we used the model of consecutive first order reactions with a reversible second step (Scheme 3) [13].

The obtained rate constant was calculated as an average value from two selected imidazole peaks resulting in *k_1_* = 9.31 × 10^−7^ s^−1^ with an R^2^ value of 0.998 for the first reaction step, and *k_2_* = 4.45 × 10^−7^ s^−1^ with an R^2^ value of 0.972. The final low level of **7** suggests that we actually observed the first order conversion from **3** to **4a**.

#### 2.2.2. Trapping the Thiiranium Intermediate **5** with AgBF_4_

Since no evidence of the thiiranium intermediate **5** during NMR reaction monitoring was found, an often-used silver salt method for thiiranium ion generation/trapping was employed.

One of the examples for this method is the use of silver tetrafluoroborate at temperatures below 0 °C [14]. To enable NMR acquisition below zero, toluene-*d_8_* was used to dissolve the “sulfur mustard” compound **3** at the temperature of −20 °C. An initial proton spectrum was recorded and then AgBF_4_ was added. Immediately after addition of the Ag-salt, a white precipitate appeared and in all subsequent proton spectra (3 h and a gradual temperature increase to 25 °C), no peaks belonging to the starting **3**, or any other unknown compounds, were detected, indicating that the formed precipitate is not soluble in toluene-*d_8_*. A small amount of DMSO-*d_6_* added at the end of the experiment dissolved the precipitate without problems.

**Figure 6:**
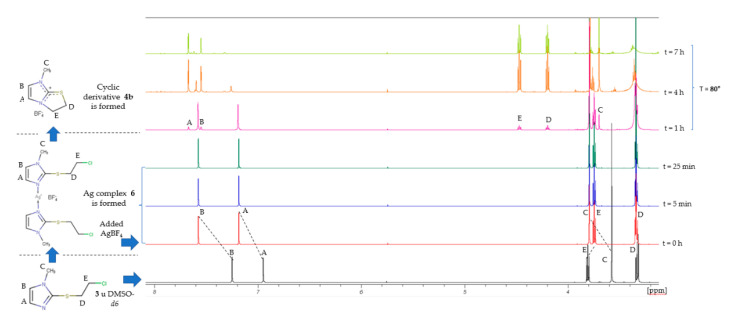
Conversion of **3** to **6** and **4b** monitored by NMR spectroscopy in DMSO-*d_6_*.

The procedure was repeated, using DMSO-*d_6_* as solvent, at 25 °C. The addition of the Ag-salt to **3** immediately gave a noticeable change in chemical shifts in the proton spectrum (Figure 6, red spectrum). Subsequently, in the preparative part of the study, this substance was synthesized and characterized as the Ag-complex of **3**, i.e., bis-{2-[(chloroethyl)thio]-1-methyl-1*H*-imidazole}-silver(I)tetrafluoroborate (**6**). However, no further change occurred during the following 40 min (Figure 6, green spectrum). By increasing the temperature to 80 °C, the reaction started and 7 h later, the signals of **6** completely disappeared, while a new set of signals indicated the formation of a new species, which was later, during the preparative procedure, discovered to be 7-methyl-2*H*, 3*H*, 7*H*-imidazo[2,1-*b*]thiazol-4-ium tetrafluoroborate (**4b**). No signals attributable to the thiiranium **5** were detected.

### 2.3. Further Synthetic Transformation

**Figure 7:**
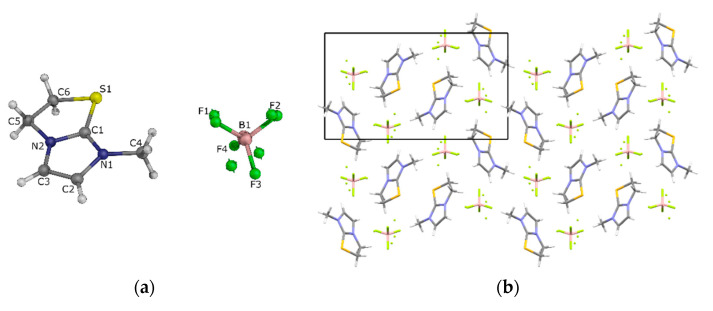
(**a**) Molecular structure of **4b** with the atomic numbering scheme (only major parts of the disordered tetrafluoroborate ion are labeled); (**b**) projection down the crystallographic *a* axis.

**Scheme 4:**
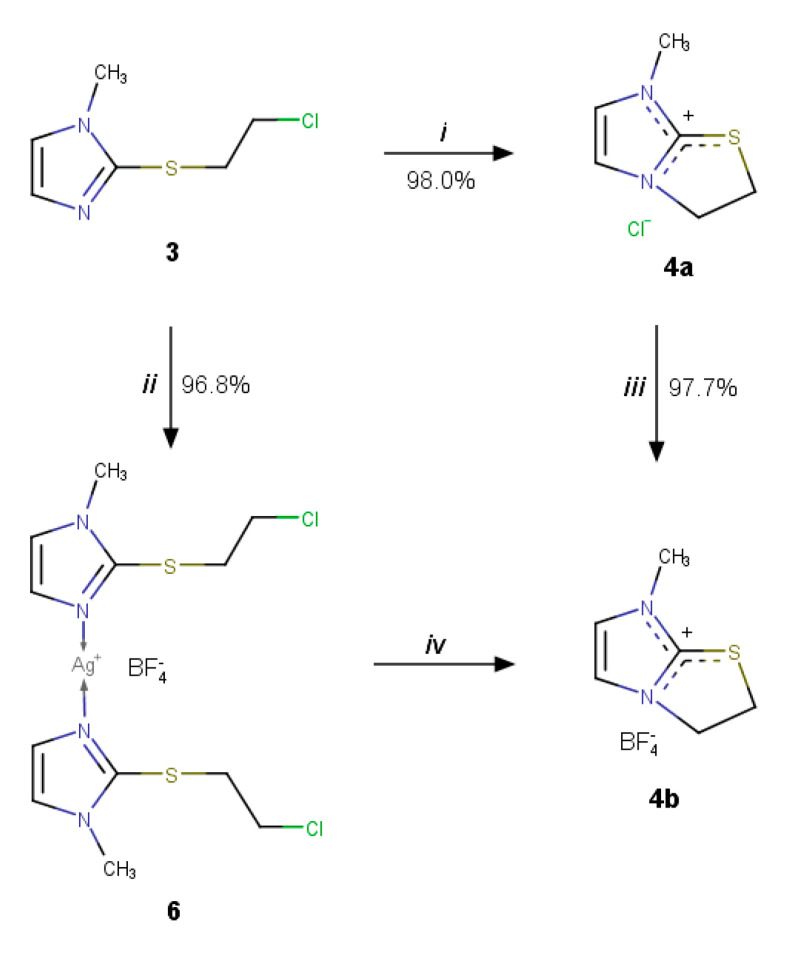
Synthetic transformation of **3** to **4a**, **6,** and **4b**; (*i*) r.t, 21 days; (*ii*) AgBF_4_, MeOH, r.t., 24 h; (*iii*) AgBF_4_, MeOH, r.t., 1 h; (*iv*) DMSO-*d_6_*, 80 °C, 7 h.

To unambiguously elucidate the structures of different forms discovered through changes in the NMR chemical shifts, **4a** was treated with silver tetrafluoborate in methanol at room temperature. After 1 hour, pure 7-methyl-2*H*, 3*H*, 7*H*-imidazo[2,1-*b*]thiazol-4-ium tetrafluoroborate (**4b**) was obtained (Scheme 4). Its structure was elucidated by NMR spectroscopy, showing similar ^1^H and ^13^C chemical shifts to the corresponding shifts of **4a** (Figure 2). Its structure was confirmed by the single crystal X-ray structure analysis (Figure 7 and Table S1).

The crystal structure of **4b** consists of planar heterobicyclic cations and tetrafluoroborate anions.

As in **4a**, the imidazole ring in **4b** exhibits significant delocalization of the electron density, while the thiazole rings are more of aliphatic character. The differences in packing of **4a** and **4b** arise from the anion coordination preferences, which are size and geometry dependable. In the absence of good hydrogen bond donors, chloride anions and tetrafluoroborate are weakly coordinated with neighboring C–H groups.

Following the same principle, treating the *S*-chloroethyl derivative **3** with silver tetrafluoborate in methanol at room temperature (Scheme 4), bis-{2-[(chloroethyl)thio]-1-methyl-1*H*-imidazole}-silver(I)tetrafluoroborate (**6**) was obtained in the form of colorless prism-shaped crystals (Scheme 4). This structure was elucidated by NMR spectroscopy and compared with the previous results from NMR measurements where heating of the Ag-complex **6** in DMSO-*d_6_* at 80 °C for 7 h led to formation of the tetrafluoroborate salt **4b.**

**Figure 8:**
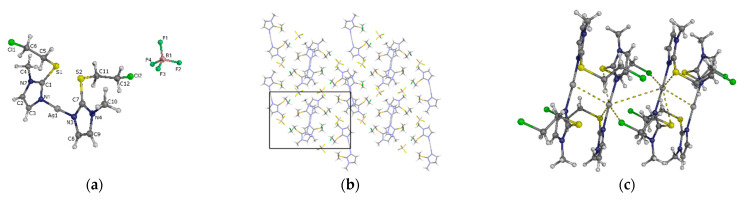
(**a**) Molecular structure of **6** with the atomic numbering scheme; (**b**) projection of **6** down the crystallographic *a* axis; (**c**) part of the crystal structure **6**: close contacts between silver(I) ions and neighboring atoms.

The structure of **6** was subsequently confirmed by the single crystal X-ray structure analysis (Figure 8 and Table S1), its main feature being a silver ion bridge between two molecules.

Silver(I) ions in **6** (Figure 8) are coordinated by two imidazole nitrogen atoms in a near-linear fashion (173.93(8)°). The imidazole rings are not symmetry related and are mutually inclined by 19° (both S-aliphatic tags are found at the same side of the cation). Interestingly, Ag1···S1 and Ag1···S2 contact lengths are shorter than the sum of the van der Waals radii, while Ag1···Cl1 (from the neighboring molecule, 2 − x, 1 − y, 1 − z) and S1···Cl1 (1 + x, y, z) are at lengths less than this sum minus 0.17 and 0.32 Å, respectively. As in **4b**, the weakly coordinating tetrafluoroborate anions are surrounded by C–H groups (Figure 8c).

Therefore, it can be concluded that **3** is not in equilibrium with the thiiranium ion **5** (pathway C, Scheme 2), but that it spontaneously transforms into the very stable compound **4a**.

**Scheme 5:**
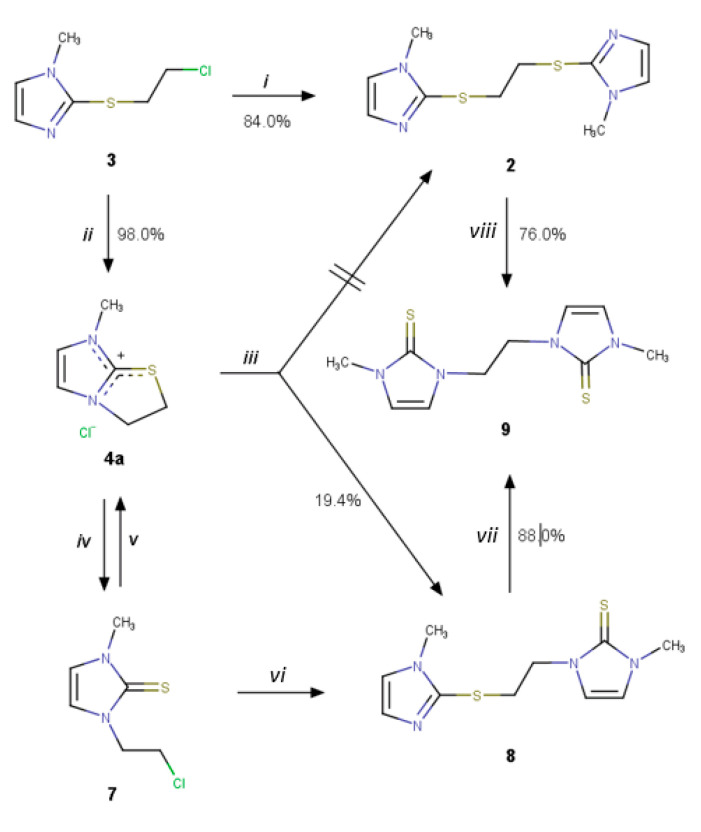
Synthetic transformation of **3**, **2**, **4a**, **7**, **8**, and **9** and their interrelations; (*i*) **1**, MeCN, HCOOH, reflux, 43 hrs; (*ii*) r.t, 21 h; (*iii*) **1**, MeCN, reflux, 10 hrs; (*iv*) MeCN, reflux, 10 hrs; (*v*) spontaneous, r.t, 18 hrs; (*vi*) **1**, MeCN, reflux, 8 hrs; (*vii*) 170 °C, 10 hrs; (*viii*) 170 °C, 17 hrs.

**Figure 9:**
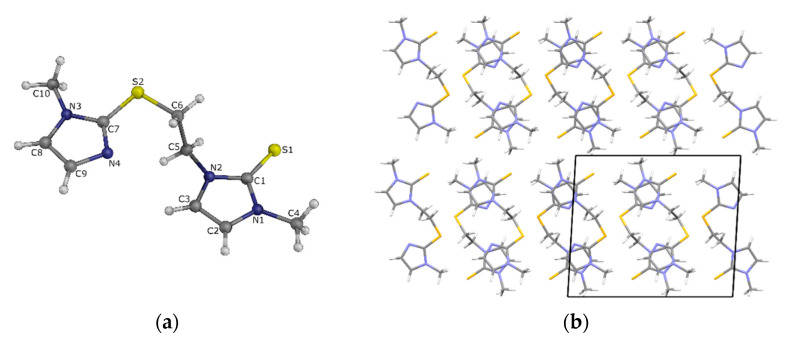
(**a**) Molecular structure of **8** with the atomic numbering scheme; (**b**) projection of **8** down the crystallographic *b* axis.

Additionally, our attempt to directly prepare **2** by reaction of **4a** with methimazole (**1**) (pathway B, Scheme 2) failed. By refluxing **4a** with **1** in acetonitrile followed by column chromatography, pure 2,3-dihydro-3-methyl-1-[(1-methyl-1*H*-imidazole-2-yl)thioethyl]-1*H*-imidazole-2-thione (**8**) was obtained in a 19.4% yield (Scheme 5). Its structure was elucidated by NMR spectroscopy and confirmed by the single crystal X-ray structure analysis (Figure 9 and Table S1). The low yield is attributed to isomerization of **7** to **4a**.

Similarly, the thione **8** was also obtained directly, in high yield (98.0%), by the reaction of **7** with **1** (Scheme 4) under reflux in MeCN.

In the crystal structure of **8,** the molecule consists of two methyl-imidazole rings separated by a thioether –CH_2_–CH_2_–S– spacer. The two rings are planar and are inclined at 6.8° (Figure 9). The bond lengths and angles are comparable to those found in the CSD database. The molecules are stacked along the crystallographic *b* axis and held together by weak hydrogen bonds of the C–H···N type and π-interactions. In the other two directions there are only weak C–H···S and H···H contacts (Figure 9b).

Furthermore, according to TLC of the reaction mixture (dichloromethane: methanol: formic acid = 8:1:0.5), no traces of **2** at Rf = 0.49 were detected. However, by using dichloromethane:acetone = 8:2 mixture as the mobile phase, beside the spot of **8** at Rf = 0.23, traces of an unknown substance at the front line (Rf = 0.98) were detected, indicating thermal instability of **4a**.

The reaction performed under the same conditions, but without **1**, led to the formation of the previously mentioned **7** (Scheme 5). Its structure was elucidated by NMR spectroscopy, with signals identical to the signals of **7** observed during kinetic decomposition of **3**. The low yield of **7** (25.0%) was attributed to the fact that no changes in the chromatogram were observed after 4–10 h of the reaction time, indicating an equilibrium between **4a** and **7**. Additionally, the attempts to prepare suitable single crystals of **7** for X-ray analysis failed because **7** isomerized back to **4a** soon after isolation at room temperature, both in the solid state and in the solution. This corroborates our conclusions drawn from the reaction kinetics data.

The structure of **8** was additionally confirmed by thermal isomerization at 170 °C for 10 h into the known crystalline 1,2-bis(2,3-dihydro-3-methyl-1*H*-imidazole-2-thione-1-yl)ethane (**9**) [15]. In analogy to a similar process [16], **9** was also obtained by thermal isomerization of **2a**. The recorded NMR spectra of **9**, obtained by both processes, were identical and mutually corroborated the proposed structure.

Finally, by the reaction of **3** with **1** in formic acid and boiling MeCN (pathway A, Scheme 2), **2a** was obtained along with unreacted **3**. NMR and MS data of both isolated products are in line with those previously reported in the literature for **2 [4]**, and for **3** in the text above. In this reaction, no traces of **4a** due to the protonation of nitrogen in **3** inhibiting intramolecular cyclisation and formation of **4a** were observed.

The synthetic and NMR studies proved that the bis-derivative **2a** is formed by direct reaction of the *S*-chloroethyl derivative **3** with methimazole (**1**) (pathway A, Scheme 2).

### 2.4. Computational Search for the Thiiranium Ion Intermediate **5**

#### 2.4.1. Reaction without External Nucleophiles

**Figure 10:**
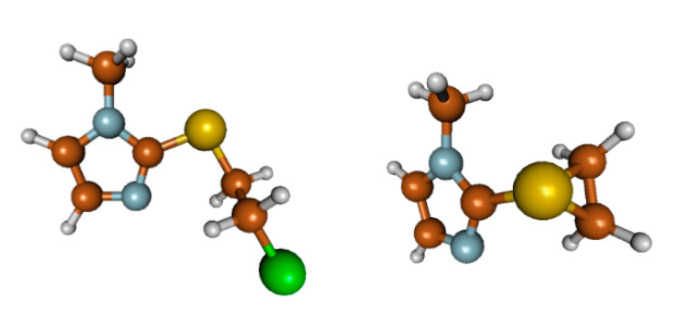
Optimized geometries of **3** (**left**) and the thiiranium cation **5** (**right**), obtained at the (SMD)/M06–2X/6–31+G(d) model.

*S*-Chloroethyl derivative **3** consists of a rigid *N*-methylimidazole ring and a flexible chloroethyl unit linked through the thioether bond (Figure 10). Its reactivity is based on the fact that the terminal C–Cl is the weakest bond in the system, which makes Cl^−^ a good leaving group. Specifically, the bond dissociation energy (BDE) that gives R–C^+^ and Cl^−^ is 25.5 kcal mol^−1^, while for the central ethyl C–C bond, it is much higher at BDE = 77.7 kcal mol^−1^ corresponding to the homolytic cleavage and offering two carbon-centered radicals. Analogously, both bonds to sulfur, C(imidazole)–S and C(alkyl)–S, are higher in energy, 73.6 and 45.4 kcal mol^−1^, respectively, and most favorably proceed via the homolytic radical pathway.

**Figure 11:**
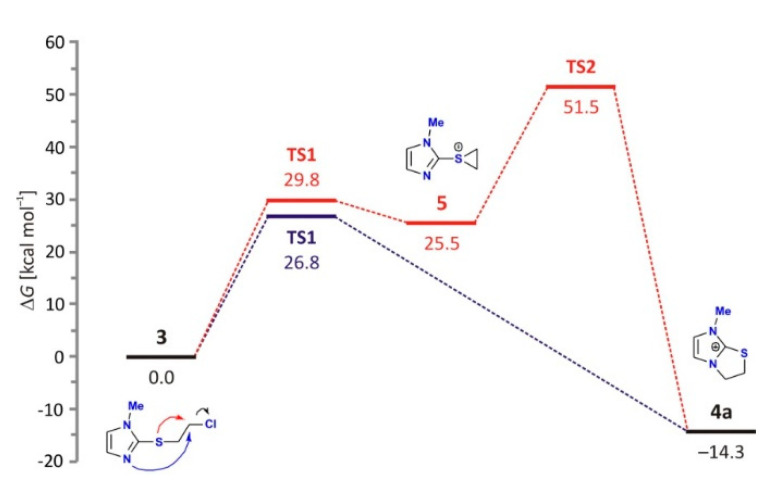
Reaction pathways for the **3** → **4a** conversion involving (i) a two-step reaction including a thiiranium ion **5** intermediate (in red), and (ii) a direct single-step process without any intermediated (in blue). Relative energies of the stationary points are obtained at the (SMD)/M06–2X/6–31+G(d) model.

The structure of **3** contains two nucleophilic centers, the S-atom and the unsaturated imidazole N-atom, and both of these can undergo an internal S_N_2 rearrangement that liberates Cl^−^ (Figure 11). The reaction with the S-atom gives the anticipated thiiranium ion **5** (Figure 11), yet the process has a very high kinetic requirement (Δ*G*^‡^ = 29.8 kcal mol^−1^), and is, following the Cl^−^ departure, thermodynamically very endergonic (Δ*G*_R_ = 25.5 kcal mol^−1^), which makes **5** an unlikely end product. From there, it can further rearrange to **4a**, yet this increases the reaction barrier to Δ*G*^‡^ = 51.5 kcal mol^−1^, despite gaining –14.3 kcal mol^−1^ in the reaction free energy, thereby rendering it unfeasible. Such a reaction profile confirms the unstable, high-energy, short-lived nature of **5**, and the inability of NMR and MS techniques to detect it. In addition, following its generation, the reaction would preferably return to **3**, rather than proceed to **4a**, which disagrees with the formation of the latter. Hence, this rules out the option that the **3** → **4a** conversion involves the thiiranium ion intermediate **5**. Alternatively, the latter process can proceed through a direct nucleophilic attack of the unsaturated imidazole N-atom onto the C-atom bearing chlorine. In this way (Figure 11), the reaction involves a single step with the activation free energy of Δ*G*^‡^ = 26.8 kcal mol^−1^, being 3.0 kcal mol^−1^ lower than required to generate **5**. This indicates a much more favorable route without the need for any intermediates, while a reasonable kinetic barrier and a significant exergonicity jointly confirm the spontaneous nature of this process under experimental conditions.

The observed differences in the reactivity of N- and S-centers can be explained by considering the atomic charges in the initial **3**. The Cl-atom is well prepared to act as an anionic leaving group, seen in its negative atomic charge of −0.11 |e|. The low nucleophilicity of the S-atom is well evidenced in its positive atomic charge, +0.27 |e|, which underlines a very limited tendency to react with electrophiles. On the other hand, both imidazole nitrogens bear negative charges, −0.42 |e| on the amine, and −0.59 |e| on the imine. While the nucleophilicity of the former is hindered by steric limitations, an even higher anionic charge on the latter rationalizes the demonstrated feasibility of the **3** → **4a** conversion involving this site.

In addition, experiments reveal a very slow further isomerization of **4a** into the *N*-(chloroethyl) derivative **7** in low concentrations. This reaction is mediated by the Cl^−^ anions that open the formed five-membered ring and offer the *N*-alkylated product. In doing so, Cl^−^ attacks the C(alkyl)–S bond in **4a** through the activation barrier of Δ*G*^‡^ = 26.1 kcal mol^−1^, which is, interestingly, 0.7 kcal mol^−1^ lower than needed to generate **4a** from **3**. However, a more significant progress of this reaction is hindered by the unfavorable reaction free energy, which for the **4a** → **7** process is endergonic at Δ*G*_R_ = 1.4 kcal mol^−1^. This nicely explains why this reaction occurs very slowly at room temperature and confirms that higher yields of **7** can be achieved in this way by increasing the reaction temperature. In other words, the conversion of **3** into compounds **4a** and **7** results in a 10-fold predominance of **4a**, because both reactions in the **3** → **4a** → **7** sequence are linked with similar kinetic requirements, yet the process leading to **4a** is significantly more favorable through a highly exergonic thermodynamic character.

The presented results lead us to conclude that the anticipated intermediate **5** makes a reasonable assumption, yet its eventual formation strongly depends on the structure of the initial reactant. Namely, systems where there are no nucleophilic centers other than the S-atom that can initiate the β-chloride elimination, make such a pathway more likely. As an illustrative example, Finn and co-workers [17] reported the nucleophilic substitution of Cl-atoms in 2,6-dichloro-9-thiabicyclo[3.3.1]nonane with external nucleophiles that proceed through a highly reactive thiiranium ion, bearing a lot of steric strain that further facilitates the reaction progress. Yet, in **3**, the presence of much nucleophilic imidazole nitrogen directs the rearrangement reaction to this site without the need for additional intermediates.

#### 2.4.2. Reaction with the Introduction of External Nucleophile

**Figure 12:**
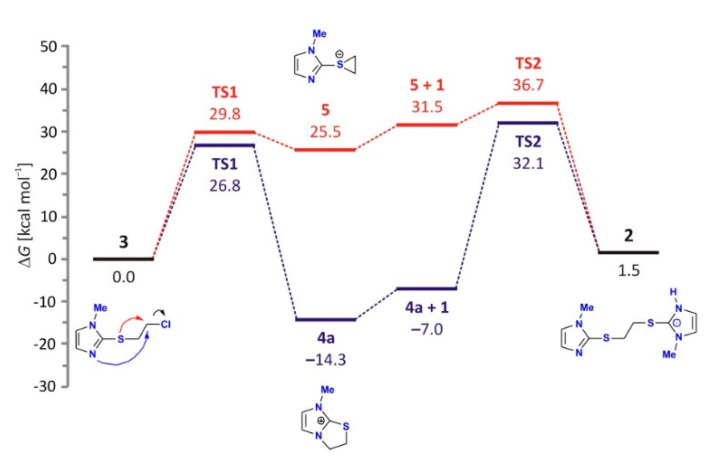
Reaction profiles for the conversion of **3** into **2** with the external nucleophile **1** proceeding through (i) a thiiranium ion **5** intermediate (in red), and (ii) a five-membered **4a** intermediate (in blue). Relative energies of stationary points are obtained at the (SMD)/M06–2X/6–31+G(d) model.

Once methimazole **1** is added to the solution of **3**, it changes the reaction outcomes by leading the conversion of the latter into **2**, isolated in the form of the HCl salt (Scheme 1). There, **1** acts as an external nucleophile, whose structure is dominated by the N–H tautomer, while its S–H analogue is 14.1 kcal mol^−1^ less stable. During the course of the reaction, **1** has the option to offer **2** either by reacting with (i) the initial **3**, (ii) the five-memebered derivative **4a**, and (iii) the three-membered derivative **5** (Scheme 2). Given the demonstrated feasibility and the spontaneous character of the **3** → **4a** conversion, it is reasonable to expect that the formation of **2** will involve **4a** as an intermediate. Indeed, following the formation of **4a** (Figure 12), it takes 7.3 kcal mol^−1^ to add **1** into the reactive complex, and an additional 39.1 kcal mol^−1^ to reach the transition state that describes the N–C cleavage within the five-membered ring and the formation of a new C–S bond with **1** that allows **2**. This becomes the rate-limiting step with an extensive activation barrier of ΔG^‡^ = 46.4 kcal mol^−1^, making such a pathway as highly unlikely. The reason for such an unfavorable reaction profile resides in the extensive stability of **4a**, –14.3 kcal mol^−1^ from the initial **3**, which disfavors its further conversions. Therefore, we can confidently state that **2** does not form from the preceding five-membered isomer **4a**.

As an alternative route, we again considered the possibility for the generation of the short-lived intermediate **5**, which could offer **2** through a reaction with **1** (Figure 12). As shown, once **2** is formed, it resides 25.5 kcal mol^−1^ higher in energy than **3**, from which it takes 6 kcal mol^−1^ to introduce **1** and further 5.2 kcal mol^−1^ to arrive at the transition state for the formation of **2**. This leads to the overall activation barrier of ΔG^‡^ = 36.7 kcal mol^−1^, which is, interestingly, 9.7 kcal mol^−1^ lower than when the reaction occurs through **4a**. Although more favorable, such a reaction profile still indicates that **5** will prefer going back to the initial **3**, rather than progressing to **2**, as the former pathway is both kinetically and thermodynamically more favorable. Therefore, this scenario also cannot explain the formation of **2** as experimentally demonstrated.

Lastly, **1** can result in **2** through a direct reaction with **3**, a process where the S-atom in **1** acts as a nucleophile that cleaves the C–Cl bond in **3** and allows **2** with the liberation of Cl^−^. This is a one-step process, which requires 8.6 kcal mol^−1^ to bring both reactants into a reactive complex for a total activation barrier of ΔG^‡^ = 33.9 kcal mol^−1^, and a reaction free energy of ΔG_R_ = 1.5 kcal mol^−1^. The calculated parameters indicate the most feasible route to generate **2**, yet the obtained kinetic and thermodynamic features make it less favorable than the internal cyclization **3** → **4a** occurring without **1**. This suggests that the generation of **2** will be slower than the reorganization to **4a**, and its higher yields will be hindered by the excess generation of the latter. However, if the reaction between **1** and **3** occurs under the acidic conditions (formic acid employed here), such an environment will convert **3** into its protonated form **3H**^+^, with excess protons residing at the unsaturated imidazole nitrogen. To support that, the basicity of this position is, on relative scales, around 10 p*K*_a_ units higher than the basicity of the S-atom in **1**. The latter quantitatively agrees with the fact that, for example, the related *N*-methylimidazole (p*K*_a_ = 6.95) [18,19] is almost 9 p*K*_a_ units more basic than *N*-methylthiourea (p*K*_a_ = −1.75) [20,21], which validates these calculations. This process will reduce the nucleophilicity of the former, which will disfavor its rearrangement to **4a**, thus allowing the only possible **3** + **1** → **2** conversion without byproducts **4a**, **7,** and **8,** thereby placing computational results in excellent agreement with experiments.

**Scheme 6:**
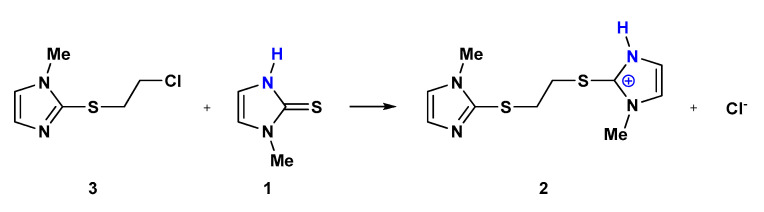
Chemical reaction between **3** and **1** liberates the Cl^−^ anion and offers monocationic **2**, which can potentially deprotonate at the secondary imidazole amine position (in blue).

In concluding this discussion, we note that the reaction between **1** and **3** gives monocationic **2** that is ΔG_R_ = 1.5 kcal mol^−1^ higher in energy than initial reactants (Figure 12). This prompted us to inspect the possibility that the formed **2** spontaneously deprotonates into the neutral system (Scheme 6), while the liberated proton joins with the chloride anion depart as HCl. Yet, such an outcome is by as much as 10.5 kcal mol^−1^ less stable, which ties in with experiments and confirms why **2** crystallizes as a hydrochloride salt, and not as, for example, an uncharged system. Finally, we investigated whether the available Cl^−^ anions can interact with **1**, thereby allowing HCl and the anionic **1^−^**, the latter potentially more nucleophilic and likely further promoting any of the studied processes. However, our calculations show that a reaction **1** + Cl^−^ → **1^−^** + HCl is exceedingly endergonic, ΔG_R_ = 28.5 kcal mol^−1^, making this possibility as highly unfeasible.

## 3. Materials and Methods

### 3.1. General

All solvents were distilled before use. Methimazole 99% was purchased from CU Chemie Ueticon, Lahr, Germany (water content 0.4%), 1,2-dichloroethane (Fisher Scientific, Bishop Meadow Road, Loughborough, UK), triethylamine (Sigma Aldrich, St. Louis, MO, USA, SAD), silver tetrafluoroborate, 99% (Alfa Aesar, Kendal. Germany), acetone (Carlo Erba Reagents, Val de Reuil Cedex, France, FRA), while formic acid, sodium sulfate anhydrous, acetonitrile, dimethylformamide, sodium chloride, sodium hydroxide, sodium hydrogen carbonate, and potassium hydroxide were purchased from Merck (Merck KGaA, Darmstadt, Germany). Solvents were distilled prior to use and dried using standard techniques. All reactions were done in flame-dried glassware. Water was purified by in-house system (Thornton 2000 CRS, Mettler Toledo, Colombus, OH, USA). pH measurements were performed using Mettler Toledo (Columbus, OH, USA) Seven Multi pH meter, and prior to measurement, it was calibrated using six points. All other used chemicals were of analytical grade. ^1^NMR and ^13^C NMR were recorded on a Bruker Avance AV600 spectrometer. High resolution mass spectra (HRMS) were recorded on an Agilent 6550 I Funnel quadrupole time-of flight mass spectrometer (QTOF) equipped with dual AJS ESI source (Agilent Technologies, Palo Alto, CA, USA). MS spectra were acquired on an Agilent 6460 triple quad (QQQ) mass spectrometer equipped with AJS ESI source (Agilent Technologies, Palo Alto, CA, USA). Ionic chromatography measurements were performed on a Thermo (Waltham, MA, USA) ionic chromatography using LC chlorine standard. HPLC analysis was performed on an Agilent Technologies (Santa Clara, CA, USA) HPLC instrument under gradient elution at a flow rate of 0.6 mL/min using mobile phase A (ammonium acetate, Merck KGaA, Darmstadt, Germany buffer) and mobile phase B (acetonitrile, Merck KGaA, Darmstadt, Germany) on a Zorbax C18 column. The effluent was monitored using the Agilent DAD/UV detector. Thermal analysis was performed using a Mettler DSC 1 instrument (Mettler Toledo, Greifensee, Swizerland) in a aluminium pans with a pierced lid at a heating rate of 10 °C/min under inert atmosphere with a flow rate of 55 mL/min. Temperature calibration was performed using the indium metal standard. Reaction progress was monitored using thin layer chromatography (NH_3_: isopropanol:toluene = 0.5:2.5:7) or (dichloromethane:acetone = 8:2) or (dichloromethane: methanol: formic acid = 8:1:0.5) on silica gel plates (Kieselgel G60 F_254,_ Merck KGaA, Darmstadt, Germany). All weighing operations were carried using Mettler Toledo (Greifensee, Switzerland) balance, daily calibrated according to the internal program.

### 3.2. NMR Spectroscopy

The complete ^1^H and ^13^C assignments were made based on one- and two-dimensional NMR spectra (^1^H, DEPTq, edited-HSQC and HMBC). All spectra were recorded on a Bruker Avance AV600 spectrometer equipped with 5 mm diameter observed probe with z-gradient accessory. All spectra were recorded using standard Bruker pulse sequences on compounds dissolved in DMSO-*d_6_*, toluene-*d_8_*, and D_2_O. Residual ^1^H and ^13^C resonances from deuterated solvent are used to reference the NMR spectra with the methyl resonance of TMS at 0.0 ppm. Variable temperature experiments were performed in the range −20 °C to 25 °C in toluene-*d_8_* and 25–80 °C in DMSO-*d_6_*.

### 3.3. Synthesis

#### 3.3.1. Reaction of Methimazole in 1,2-Dichloroethane at Room Temperature

Methimazole (**1**, 100 mg, 0.87 mmol) was dissolved in 10 mL of 1,2-dichloroethane. The solution was left without stirring for 15 days in the dark at room temperature in a humidity non-controlled environment. The precipitated product was collected using vacuum suction and rinsed a few times with cold 1,2-dichloroethane, yielding colorless plate-shaped crystals of 1,2-bis[(1-methyl-1*H*-imidazole-2-yl)thio]ethane dihydrochloride tetrahydrate (**2b**, 51 mg, 45.6% calc. on consumed/converted **1**) Mp. (DSC, onset) 208 °C. Melting point and other physical constants were in line with the previously reported [4].

Mother liquor was concentrated *in vacuo* to dryness and the remaining residue was purified by column chromatography on silica gel (mesh size 0.063–0.2) and dichloromethane: acetone 8:2 as eluent; pure 2-[(chloroethyl)thio]-1-methyl-1*H*-imidazole (**3**, 12 mg, 12.1% calc. on consumed **1**) was isolated in the form of colorless oil. TLC: Rf = 0.76 (dichloroethane:acetone = 8:2), Rf = 0.66 (ammonia solution, w = 25%:2-propanol:toluene = 0.5:2.5:7), MS-QTOF: m/z: [M + H]^+^ 177.0266, C_6_H_9_N_2_SCl. ^1^H NMR (600 MHz, DMSO-*d_6_*, ppm): N-CH_3_ (s, 3 H) 3.58, S-CH_2_- (d, 2 H) 3.33, *J* = 7.2 Hz, -CH_2_-Cl (d, 2H) 3.81, *J* = 7.3 Hz, =N-CH= (d, 1H) 6.95, *J* = 1.3 Hz, -N(CH_3_)-CH= (d, 1H) 7.26, *J* = 1.1 Hz. ^13^C NMR (151 MHz, DMSO-*d_6_*, ppm): N-CH_3_ 33.3, S-CH_2_-35.7, -CH_2_-Cl 43.7, =N-CH= 129.1, -N(CH_3_)-CH= 123.9, C(S) 139.6.

After isolation of **3**, by evaporation of the leftover fraction, unreacted, TLC pure, methimazole (1, 36 mg, 36.0%) was recovered. Mp. (DSC, onset) 145 °C (lit. 144–147 °C [1]) was identical to the mp. of the used starting material. So, only 64 mg of **1** were converted to the products.

#### 3.3.2. 2-[(Chloroethyl)thio]-1-methyl-1*H*-imidazole (**3**)

Methimazole (**1**, 100 mg, 0.87 mmol) and formic acid (0.12 mL, 3.18 mmol) were dissolved in 1,2-dichloroethane (10 mL) and refluxed for 17 h. The mixture was evaporated *in vacuo*, diluted with water (10 mL), neutralized with 1 M NaOH, and extracted three times with dichloromethane. Organic layer was dried over anhydrous sodium sulfate, evaporated under reduced pressure, and purified by column chromatography using silica gel (mesh size 0.063–0.2) and dichloromethane:acetone = 8:2 as eluent yielding 2-[(chloroethyl)thio]-1-methyl-1*H*-imidazole (**3**, 113 mg, 73.5%) in the form of colorless oil and 1,2-bis[(1-methyl-1*H*-imidazole-2-yl)thio] ethane in the form of a white crystalline solid (**2a**, 27 mg, 24.2%). Obtained NMR and MS data are in line with those reported previously in the literature for **2a** [4] and for **3** in the text.

#### 3.3.3. Reaction of Methimazole (**1**) in Boiling Dry 1,2-Dichlorethane

Methimazole (**1**, 1000 mg, 8.76 mmol) was dissolved in dry 1,2-dichlorethane (10 mL) and stirred under reflux for 11 h. The solvent was evaporated *in vacuo* to dryness, and the residue was triturated with cold dichloroethane; the precipitate was collected using vacuum suction, rinsed a few times with cold 1,2-dichloroethane, and dried at 102 °C for 2 h. The crude, white solid product (600 mg) comprised of, according to the NMR analysis, 7-methyl-2*H*, 3*H*, 7*H*-imidazo[2,1-*b*]thiazol-4-ium chloride (**4a**, 20.0% in mixture, 7.8%, not isolated) and 1,2-bis[(1-methyl-1*H*-imidazole-2-yl)thio]ethane dihydrochloride (**2d**, 80.0% in mixture, 33.5%). It was further subjected to fractionation by column chromatography using silica gel (mesh size 0.063–0.2) and dichloromethane:acetone = 8:2 as eluent, yielding 1,2-bis[(1-methyl-1*H*-imidazole-2-yl)thio]ethane dihydrochloride (**2d**, 480 mg, 33.5%) in the form of pure colorless solid. Melting point (DSC, onset): 217 °C, TLC: Rf = 0.60 (ammonia solution, w = 25%: 2-propanol: toluene= 0.5: 2.5: 7), MS-QTOF: [M + H-2HCl]^+^ 255.0743, [M-2HCl] 254.066, C_10_H_14_N_4_S_2_. ^1^H NMR (600 MHz, D_2_O, ppm): N-CH_3_ (s, 6H) 3.82, S-CH_2_- (s, 4H) 3.26, N-CH= (d, 2H) 7.46, *J* = 1.8 Hz, -N(CH_3_)-CH= (d, 2H) 7.50, *J* = 2.2 Hz. ^13^C NMR (151 MHz, D_2_O, ppm): N-CH_3_ 34.6, S-CH_2_- 34.0, N-CH= 120.5, -N(CH_3_)-CH= 125.1 C(S) 138.0. Purity (HPLC): 98%, RRT: 20.518.

7-Methyl-2*H*, 3*H*, 7*H*-imidazo[2,1-*b*]thiazol-4-ium chloride (**4a**) comprised of the crude product was not isolated. ^1^H NMR (600 MHz, D_2_O, ppm): N-CH_3_ (s, 3H) 3.62, S-CH_2_- (t, 2H) 4.13, *J* = 7.7 Hz, N-CH_2_ (t, 2H) 4.43, *J* = 7.7 Hz, N-CH= (d, 1H) 7.31, *J* = 2.2 Hz, -N(CH_3_)-CH= (d, 1H) 7.2, *J* = 2.2 Hz. ^13^C NMR (151 MHz, D_2_O, ppm): N-CH_3_ 35.2, S-CH_2_- 48.6, N-CH_2_ 37.1, N-CH= 118.6, -N(CH_3_)-CH= 126.2 C(S) 150.4. NMR spectra in D_2_O were in line with spectra of **4a** sample obtained by isomerization of **3**, but taken in DMSO-*d_6_* (see below).

Mother liquor that remained after suction of the crude solid product was concentrated *in vacuo*, and the crude residue was purified using column chromatography on silica gel (mesh size 0.063–0.2) and dichloromethane:acetone = 8:2 as eluent, yielding pure 2-[(chloroethyl)thio]-1-methyl-1*H*-imidazole (**3**, 800 mg, 51.7%) in the form of a colorless oil. Obtained NMR data were in line with what was previously reported in the text.

#### 3.3.4. 7-Methyl-2*H*, 3*H*, 7*H*-imidazo[2,1-b]thiazol-4-ium chloride (**4a**)

Method (A) 2-[(Chloroethyl)thio]-1-methyl-1*H*-imidazole (**3**, 100 mg, 0.56 mmol) was held for 21 days at 25 °C/60%RH. Formed crystalline product was triturated with cold dichloromethane, collected using vacuum suction, rinsed a few times with cold dichloromethane, and dried, yielding 7-methyl-2*H*, 3*H*, 7*H*-imidazo[2,1-*b*]thiazol-4-ium chloride (**4a**) in the form of needle like crystals (98 mg, 98.0%). Melting point (DSC, onset): 241 °C, TLC: Rf = 0.49 (dichloromethane: methanol: formic acid = 8:1:0.5), ESI MS-QTOF: [M-Cl]^+^ 141.0485, C_6_H_9_N_2_SCl. ^1^H NMR (600 MHz, DMSO-*d_6_*, ppm): N-CH_3_ (s, 3H) 3.72, S-CH_2_- (m, 2H) 4.16–4.26, N-CH_2_ (t, 2H) 4.50, *J* = 7.9 Hz, N-CH= (d, 1H) 7.73, *J* = 2.0 Hz, -N(CH_3_)-CH= (d, 1H) 7.62, *J* = 1.8 Hz. ^13^C NMR (151 MHz, DMSO-*d_6_*, ppm): N-CH_3_ 36.3, S-CH_2_- 39.0, N-CH_2_ 49.5, N-CH= 120.2, -N(CH_3_)-CH= 127.6 C(S) 151.1. Ionic chromatography experimentally determined the percentage of Cl ions (19%) that corresponded to the theoretical value (20%) within the experimental error. The structure of **4a** was confirmed by single crystal X-ray diffraction analysis (Figure 3 and Table S1).

Method (B) 1-(Chloroethyl)-3-methyl-1*H*-imidazole-2-thione (**7**, 50 mg, 0.28 mmol) was purified prior to analysis by column chromatography using silica gel (mesh size 0.063–0.2) and dichloromethane:acetone = 8:2 as eluent. The pure sample was dissolved at room temperature in DMSO-*d_6_* (600 µL) after 8 h and subjected to NMR analysis. According to the obtained spectra, the sample consists of 49.0% **7** and 51.0% **4a** (yield expressed in molar percentages), which indicates fast isomerization. After 18 h, only peaks of **4a** were present in the spectra, confirming complete isomerization of **7** to **4a**.

#### 3.3.5. 7-Methyl-2*H*, 3*H*, 7*H* -imidazo[2,1-b]thiazol-4-ium tetrafluoroborate (**4b**)

Method (A) 7-Methyl-2*H*, 3*H*, 7*H*-imidazo[2,1-*b*]thiazol-4-ium chloride (**4a**, 115 mg, 0.65 mmol) was dissolved in methanol (10 mL), and under stirring (126 mg, 0.65 mmol) silver tetrafluoroborate was added in portions and stirred at the room temperature for 60 min. Colloidal precipitate was filtered through 0.2 µm PTFE filter and filtrate evaporated *in vacuo* to dryness yielding 7-methyl-2*H*, 3*H*, 7*H*-imidazo[2,1-*b*]thiazol-4-ium tetrafluoroborate (**4b** 145 mg, 97.7%) in the form of colorless needle-like crystals. Melting point (DSC, onset): 135 °C, TLC: Rf = 0.55 (dichloromethane: methanol: formic acid= 8:1:0.5) EI-MS-QQQ: [M]^+^140.9. ^1^H NMR (600 MHz, DMSO-*d_6_*, ppm): N-CH_3_ (s, 3H) 3.70, S-CH_2_- (t, 2H) 4.20, N-CH_2_ (t, 2H) 4.47, N-CH= (d, 1H) 7.68 *J* = 2.2 Hz, -N(CH_3_)-CH= (d, 1H) 7.56, *J* = 1.8 Hz. ^13^C NMR (151 MHz, DMSO-*d_6_*, ppm): N-CH_3_ 35.8, S-CH_2_- 38.4, N-CH_2_ 49.0, N-CH= 119.7, -N(CH_3_)-CH= 127.1, C(S) 150.4. The structure of **4b** was confirmed by single crystal X-ray diffraction analysis (Figure 7 and Table S1).

Method (B) 2-[(Chloroethyl)thio]-1-methyl-1*H*-imidazole (**3**, 6.0 mg, 0.04 mmol) was purified prior to analysis by column chromatography using silica gel (mesh size 0.063–0.2) and dichloromethane:acetone = 8:2 as eluent. Pure sample was dissolved at room temperature in DMSO-*d_6_* (600 µL) and to that solution silver tetrafluoroborate (6.0 mg, 0.04 mmol) was added and the solution heated at 80 °C for 7 h. According to the obtained spectra, the sample consists of 98.0% of **7** (yield expressed in molar percentage). NMR data were in line with the data for **4b** reported previously under method A.

#### 3.3.6. Bis-{2-[(chloroethyl)thio]-1-methyl-1*H*-imidazole}-silver(I) Tetrafluoroborate (**6**)

2-[(Chloroethyl)thio]-1-methyl-1*H*-imidazole (**3**, 40 mg, 0.23 mmol) was dissolved in methanol (7 mL) and to that solution silver tetrafluoroborate (44 mg, 0.23 mmol) was added in portions under nitrogen and stirring at room temperature, and stirred for an additional 4 h at room temperature (22 °C). The reaction mixture was left overnight at room temperature; the precipitated product was collected using vacuum suction and rinsed a few times with cold 1,2-dichloroethane (15 mL), yielding colorless prism-shaped crystals of bis-{2-[(chloroethyl)thio]-1-methyl-1*H*-imidazole}-silver(I)tetrafluoroborate (**6**, 61 mg, 96.8%). Mp. (DSC, onset): 133 °C. ^1^H NMR (600 MHz, DMSO-*d_6_*, ppm): N-CH_3_ (s, 6H) 3.79, S-CH_2_- (t, 4H) 3.35, CH_2_-Cl (t, 4H) 3.75, N-CH= (d, 2H) 7.57, -N(CH_3_)-CH= (d, 2H) 7.19. The structure of **6** was confirmed by single X-ray diffraction analysis (Figure 8 and Table S1).

#### 3.3.7. Reaction of 2-[(Chloroethyl)thio]-1-methyl-1*H*-imidazole (**3**) with Methimazole (**1**)

2-[(Chloroethyl)thio]-1-methyl-1*H*-imidazole (**3**, 50 mg, 0.28 mmol) and methimazole (**1**, 33 mg, 0.28 mmol) were dissolved in acetonitrile (7 mL), and formic acid (w =37%, 0.0287 mL, 0.28 mmol) was added under stirring, and refluxed for 43 h. The mixture was evaporated *in vacuo*, diluted with water (7 mL), neutralized with 1 M NaOH, and extracted three times with dichloromethane. Organic layer was dried over anhydrous sodium sulfate, evaporated under reduced pressure, and purified by column chromatography using silica gel (mesh size 0.063–0.2) and dichloromethane:acetone = 8:2 as eluent yielding 1,2-bis[(1-methyl-1*H*-imidazole-2-yl)thio]ethane in the form of white crystalline solid (**2a**, 18 mg, 84.0% calc. on reacted **3**) and unreacted 2-[(chloroethyl)thio]-1-methyl-1*H*-imidazole (**3**, 35,1 mg, 70.2%). The obtained NMR and MS data are in line with the previously reported data for **2a** [4] and for **3** in the text.

#### 3.3.8. 1-Chloroethyl-2,3-dihydro-3-methyl-1*H*-imidazole-2-thione (**7**)

7-Methyl-2*H*, 3*H*, 7*H*-imidazo[2,1-*b*]thiazolinium chloride (**4a**, 100 mg, 0.56 mmol) was dissolved in acetonitrile (10 mL) and heated under reflux. No changes in the chromatogram were observed after 5 h of heating, hence indicating the formation of an equilibrium between **4a** and the product. The mixture was heated in total for 10 h, diluted with water (10 mL), and brine extracted with dichloromethane. The organic layer was dried under anhydrous sodium sulfate, evaporated under reduced pressure yielding TLC pure 1-chloroethyl-2,3-dihydro-3-methyl-1*H*-imidazole-2-thione (**7**, 25 mg, 25.0%) in the form of colorless crystals. Melting point (DSC, onset): 65 °C. TLC: Rf = 0.97 (dichloromethane: acetone= 8: 2). EI-MS-QQQ: [M + H]^+^ 177,6. ^1^H NMR (600 MHz, DMSO-*d_6_*, ppm): N-CH_3_ (s, 3H) 3.47, CH_2_-Cl (t, 2H) 3.96, *J* = 6.1, N-CH_2_ (t, 2H) 4.27, *J* = 5.9 Hz, =CH-N-CH_3_ (d, 1H) 7.16, *J* = 2.6 Hz, CH_2_-N-CH= (d, 1H) 7.20, *J* = 2.2 Hz,. ^13^C NMR (75 MHz, DMSO-*d_6_*, ppm): N-CH_3_ 34.3, CH_2_-Cl 41.7, N-CH_2_ 48.4, =CH-N-CH_3_ 118.2, CH_2_-N-CH= 118.0, C(S) 161.9.

#### 3.3.9. 2,3-Dihydro-3-methyl-1-[(1-methyl-1*H*-imidazole-2-yl)thioethyl]-1*H*-imidazole-2-thione (**8**)

Method (A): A solution of 7-methyl-2*H*, 3*H*, 7*H*-imidazo[2,1-*b*]thiazol-4-ium chloride (**4a**, 100 mg, 0.56 mmol) and methimazole (**1**, 64 mg, 0.56 mmol) in acetonitrile (10 mL) was refluxed for 10 h. The mixture was evaporated under reduced pressure to dryness, the residue dissolved in water (10 mL), neutralized with 1 M NaOH, and extracted with dichloromethane. The organic layer was dried under anhydrous sodium sulphate, concentrated to dryness, and the residue purified by column chromatography on silica gel (mesh size 0.063–0.2) using dichloromethane:acetone = 8:2 as eluent. After evaporation of the selected fraction under reduced pressure, TLC pure 2,3-dihydro-3-methyl-1-[(1-methyl-1*H*-imidazole-2-yl)thioethyl]-1*H*-imidazole-2-thione (**8**, 28 mg, 19.4%) was obtained in the form of white crystalline product. The reason for the low yield is the formation of an equilibrium between **4a** and **7**, and consequently only approx. 20–25% of **7** can react with **1**. Melting point (DSC, onset): 101.7 °C, TLC: Rf = 0.23 (dichloromethane: acetone= 8:2), Rf = 0.61 (dichloromethane: methanol: formic acid= 8:1:0.5), EI-MS-QQQ: m/z [M + H]^+^ 255.0, ^1^H NMR (600 MHz, DMSO-*d_6_*, ppm): N-(CH_3_)-C-S- (s, 3H) 3.58, S-CH_2_- (t, 2H) 3.31, *J* = 6.6 Hz, C(S)-N-CH_3_ (s, 3H) 3.46, N-CH_2_ (t, 2H,) 4.15, *J* = 6.6 Hz, N-(CH_3_)-CH= (d, 1H) 7.11, *J* = 2.2 Hz, CH-N-CH_2_ (d, 1H,) 7.17, *J* = 2.2 Hz, CH-N= (d, 1H) 6.97, *J* = 1.1 Hz, CH-N-CH_3_ (d, 1H) 7.27, *J* = 1.5 Hz. ^13^C NMR (151 MHz, DMSO-*d_6_,* ppm): C(S)-N-CH_3_ 34.3, S-CH_2_ 31.7, N-(CH_3_)-C-S- 32.9, N-CH_2_ 46.1, N-(CH_3_)-CH= 118.1, CH-N-CH_2_ 117.7, CH-N= 128.6, CH-N-(CH_3_)-C-S- 123.5, C-S-CH_2_- 138.1, C(S)161.4. The structure was confirmed by single crystal X-ray diffraction analysis (Figure 9 and Table S1).

Method (B): 1-Chloroethyl-2,3-dihydro-3-methyl-1*H*-imidazole-2-thione (**7**, 100 mg, 0.56 mmol) with **1** (64 mg, 0.56 mmol) was refluxed in MeCN for 8 h. The mixture was evaporated under reduced pressure to dryness, the residue dissolved in water (10 mL), neutralized with 1 M NaOH, and extracted with dichloromethane. The organic layer was dried under anhydrous sodium sulphate, concentrated to dryness, and the residue purified by column chromatography on silica gel (mesh size 0.063–0.2) using dichloromethane: acetone = 8:2 as eluent. After evaporation of the selected fraction under reduced pressure, TLC pure 2,3-dihydro-3-methyl-1-[(1-methyl-1*H*-imidazole-2-yl)thioethyl]-1*H*-imidazole-2-thione (**8**, 139 mg, 98.0 %) was obtained in the form of white crystalline product. Obtained melting point, Rf value, and NMR data are in line with the data reported previously in the text for **8**.

#### 3.3.10. 1,2-Bis(2,3-dihydro-3-methyl-1*H*-imidazole-2-thione-1-yl)ethane (**9**)

Method (A): 1,2-Bis[(1-methyl-1*H*-imidazole-2-yl)thio]ethane (**2a**, 50 mg, 0.20 mmol) was heated in sand bath at the temperature of 170 °C for 17 h. The melt was dissolved in a mixture of dichloromethane: acetone= 8:2 and fractioned by column chromatography on silica gel (mesh size 0.063–0.2) using dichloromethane: acetone 8:2 as eluent. After evaporation of the selected fraction under reduced pressure, TLC pure red crystalline 1,2-Bis(2,3-dihydro-3-methyl-1*H*-imidazole-2-thione-1-yl)ethane (**9**, 38 mg, 76.0%) was obtained. Melting point (DSC, onset): 195 °C (Lit.: mp. 194–195 °C) [14]. TLC: Rf = 0.59 (dichloromethane:acetone = 8:2), Rf = 0.76 (dichloromethane: methanol: formic acid= 8:1:0.5), EI-MS-QTOF: 254.37. ^1^H NMR (600 MHz, DMSO-*d_6_*, ppm): N-CH_3_ (s, 6H), 3.45, -N(CH_3_)-CH= (d, 2H) 7.08, *J* = 2.2 Hz, CH_2_-N-CH= (d, 2H) 6.83, *J* = 2.2 Hz, N-CH_2_ (s, 4H) 4.31. ^13^C NMR (151 MHz, DMSO-*d_6_*, ppm): N-CH_3_ 34.4, -N(CH_3_)-CH= 118.4, CH_2_-N-CH= 117.2, N-CH_2_ 45.1, C(S) 161.7.

Method (B): 2,3-Dihydro-3-methyl-1-[(1-methyl-1*H*-imidazole-2-yl)thioethyl]-1*H*-imidazole-2-thione (**8**, 50 mg, 0.20 mmol) was heated in a sand bath at 170 °C for 10 h. The melt was dissolved in the mixture of dichloromethane:acetone = 8:2 and fractioned by column chromatography on silica gel (mesh size 0.063–0.2) using dichlormethane: acetone = 8:2 as eluent. After evaporation of the selected fraction under reduced pressure, TLC pure red crystalline 1,2-Bis(2,3-dihydro-3-methyl-1*H*-imidazole-2-thione-1-yl)ethane (**9**, 44 mg, 88.0%) was obtained. The obtained melting point, Rf value, and NMR data are in line with the data reported previously in the text for **9**.

### 3.4. NMR Search for the Thiiranium Intermediate **5**

#### 3.4.1. Kinetics of 2-[(Chloroethyl)thio]-1-methyl-1*H*-imidazole (**3**) Isomerization in DMSO-*d_6_*

2-[(Chloroethyl)thio]-1-methyl-1*H*-imidazole (**3**, 30 mg, 0.2 mmol) was dissolved in DMSO-*d_6_* (600 µL) to the final concentration of 0.3 M and the course of the reaction was monitored by ^1^H NMR spectroscopy over the period of 3 months at 25 °C. Comparison of the first proton spectrum taken immediately after dilution and ones taken after 3 days showed no differences. Spectra were taken periodically to the almost complete (99.1%) conversion of **3** to the **4a**. A minor quantity of **7** was also detected (approx. 0.94%). The obtained NMR data for **4a** and **7** are in line with the data reported previously in the text. The proton spectra were processed and the rate constants calculated using Dynamics Center 2.6.3 software.

#### 3.4.2. Search for Thiiranium Intermediate **5** in the Reaction of 2-[(Chloroethyl)thio]-1-methyl-1*H*-imidazole **3** with Silver Tetrafluoroborate in Toluene-*d_8_*

2-[(Chloroethyl)thio]-1-methyl-1*H*-imidazole (**3**, 6.0 mg, 0.04 mmol) was dissolved in toluene-*d_8_* (600 μL), the solution cooled down to −20 °C, and the proton NMR spectrum of the solution was recorded at the same temperature, showing the signals of **3** accompanied by solvent signals. Then, silver tetrafluoroborate (6.0 mg, 0.04 mmol) was added and a series of consecutive proton NMR spectra were recorded for a total duration of 3 h while the temperature was gradually increased from −20 °C to 25 °C. Immediately after the addition of the salt, a white precipitate appeared and in all proton spectra no peaks belonging to the starting three were noticed, indicating that the formed precipitate is not soluble in toluene-*d_8_*. After no new signals appeared in a period of 3 h and the temperature reached 25 °C, DMSO-*d_6_* (600 μL) was added, the precipitate dissolved, and some new peaks appeared.

#### 3.4.3. Search for the Thiiranium Intermediate **5** in the Reaction of 2-[(Chloroethyl)thio]-1-methyl-1*H*-imidazole **3** with Silver Tetrafluoroborate in DMSO-*d_6_*

2-[(Chloroethyl)thio]-1-methyl-1*H*-imidazole (**3**, 6.0 mg, 0.04 mmol) was dissolved in DMSO-*d_6_* (600 μL) at 25 °C and the proton NMR spectrum of the solution was recorded showing the signals of **3**. After the addition of silver tetrafluoroborate (6.0 mg, 0.04 mmol), the recorded ^1^H spectrum at the same temperature showed the signals belonging to Ag-complex **6**. No other signals were noticed. After that, the temperature was increased up to 80 °C and the NMR spectra were consecutively recorded for a total duration of 7 h. After 1 h at 80 °C, the recorded proton spectrum exhibited low intensity signals belonging to tetrafluoroborate salt **4b** in addition to the resonance lines of **6**. Four hours at 80 °C later, the signals of **6** completely disappeared while signals of **4b** dominated. Transformation from **3** to **4b** was expected as the equimolar quantities of **3** and silver tetrafluoroborate were used, and the performed experiment clearly confirmed transformation of **3** via **6** to **4b** (Figure 6).

### 3.5. Single Crystal X-ray Diffraction

Single crystals were obtained by the following methods: **4a** by spontaneous isomerization of **3** followed by crystallization, **4b** and **6** by slow evaporation from methanol, and **8** by slow evaporation from water.

The crystal and molecular structures of **4a**, **4b**, **6**, and **8**, were determined by single-crystal X-ray diffraction at the following temperatures: 293 K for **4a**, 170 K for **4b**, **6** and **8** (Table S1). Single-crystal diffraction experiments were performed on an Oxford Diffraction Xcalibur diffractometer (**4a**) and on a Rigaku Oxford Diffraction XtaLAB Synergy-S diffractometer (**4b**, **6** and **8**). Program package CrysAlisPro [22] was used for data collection, cell refinement, and data reduction. Structures were solved by direct methods and refined using the SHELXT [23] and SHELXL [24] programs, respectively. The refinement procedure by the full-matrix least squares methods based on *F*^2^ values against all reflections included anisotropic displacement parameters for all non-H atoms. The positions of H-atoms riding on carbon atoms were determined on stereochemical grounds. The anion moiety in **4b** was modelled using various restraints (DFIX, SADI, SIMU and ISOR). The SHELX programs operated within the Olex2 crystallographic suite. Geometrical calculations and molecular graphics were done with MERCURY [25]. Supplementary crystallographic data sets for the structures are available through the Cambridge Structural Data base with deposition numbers 2,105,605–2,105,608. A copy of this information may be obtained free of charge from the director, CCDC, 12 Union Road, Cambridge, CB2 1EZ, UK (fax: +44-1223-336-033; e-mail: deposit@ccdc.cam.ac.uk or http://www.ccdc.cam.ac.uk.

### 3.6. Computational Methods

All molecular geometries were optimized with a very efficient M06–2X/6–31+G(d) model, which was designed to provide highly accurate thermodynamic and kinetic parameters for various organic systems. To account for the solvent effects, during geometry optimization, the implicit SMD solvation model corresponding to pure DMSO was included. Thermal corrections were extracted from the corresponding frequency calculations, so that all of the presented results correspond to differences in the Gibbs free energies at room temperature and normal pressure. The choice of such computational setup was prompted by its success in reproducing various features of different organic [26,27], organometallic [28,29], and enzymatic systems [30,31], being particularly accurate for relative trends among similar reactants, which is the focus here. All transition state structures were located using the scan procedure, employing both 1D and 2D scans, the latter specifically utilized to exclude the possibility for concerted mechanisms. Apart from the visualization of the obtained negative frequencies, the validity of all transition state structures was confirmed by performing IRC calculations in both directions and identifying the matching reactant and product structures connected by the inspected transition state. All the calculations were performed using the Gaussian 16 software [32].

## 4. Conclusions

**Scheme 7:**
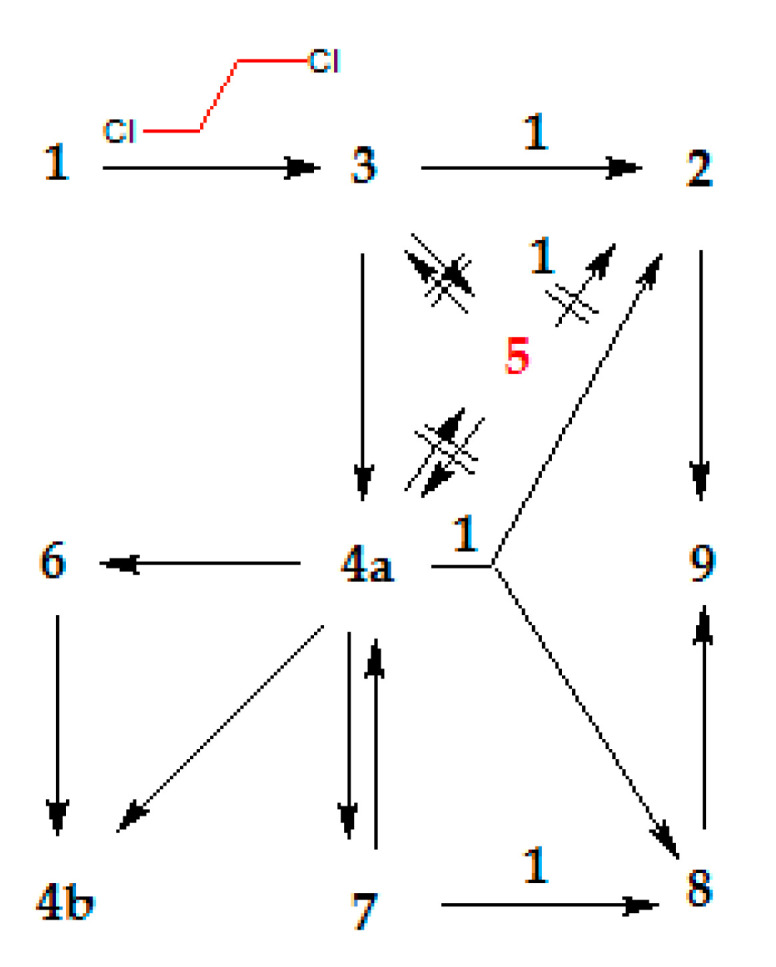
Synthetic interconversion and isomerisation involving **4a**.

We established that spontaneous transformation of methimazole (**1**) with 1,2-dichloroethane to **2** under mild conditions proceeds by the direct attack of **1** at the chloroethyl derivative **3**. Although the isomerization of **3** into the stable thiiranium isomer **4a** was observed (Scheme 7), the **1** to **2** conversion does not follow that pathway, nor does it go through the thiiranium ion **5,** postulated in analogy to similar reactions reported in literature.

The imidazothiazolium salt **4a** in reaction with **1** prefers isomerization to the *N*-chloroethyl derivative **7**, rather than alkylation to **2.** The **7** further reacts with **1** and forms dimeric thione **8** in low yield. The structures of both **2** and **8** were confirmed by their thermal isomerization to **9**. The intermediate thiiranium ion **5** was not detected by chromatographic and spectroscopic methods, nor caught by trapping it with AgBF_4_. However, during the trapping reaction, the silver complex of **3***,* i.e., **6,** was isolated. When heated to 80 °C, **6** cyclized into the tetrafluoroborate salt **4b.**

Conclusions from the computational studies, are in agreement with our experimental results, showed that the bis-derivative **2** is formed by direct reaction of **3** with **1**, rather than through the postulated, but unlikely, thiiranium ion **5** or *via* the stable imidazotiazolium salt **4a**.

Caution is needed when handling methimazole, due to its high reactivity in 1,2-dichoroethane solution, even under mild conditions, and we discussed this previously. Our current results additionally revealed that in the **1** to **2** conversion, a “sulfur mustard” type compound **3** is also created, which is a further warning that the use of 1,2-dichloroethane should be avoided when working with methimazole.

## Data Availability

Not applicable.

## Supplementary Materials

The following are available online, Table S1: Crystal data and structure refinement for **4a**, **4b**, **6** and **8**.
