# Supplementary material for: Chemistry of Spontaneous Alkylation of Methimazole with 1,2-Dichloroethane
Source: Molecules. 2021 Nov 21;26(22):7032. doi: 10.3390/molecules26227032 (PMC8625577; doi:10.3390/molecules26227032)
Supplement: Supplementary file 1 [file molecules-26-07032-s001.zip › molecules-1437185-supplementary.pdf]

Supplementary Information:

Chemistry of Spontaneous Alkylation of Methimazole with 1,2-Dichloroethane

Leo Štefan, Ana Čikoš, Robert Vianello, Ivica Đilović, Dubravka Matković-Čalogović and Miljenko Dumić

Table S1. Crystal data and structure refinement for **4a**, **4b**, **6** and **8.**

	**4a**	**4b**	**6**	**8**
Molecular formula	$[C_6H_9N_2S]Cl$	$[C_6H_9N_2S]BF_4$	$[(C_6H_9ClN_2S)_2Ag]BF_4$	$C_{10}H_{14}N_4S_2$
Formula weight	176.66	228.02	548.00	254.37
Temperature/K	293(2)	169.99(10)	169.99(10)	169.99(10)
Crystal system	Orthorhombic	Monoclinic	Monoclinic	Monoclinic
Space group	*Pnma*	*P*2₁/*c*	P2₁/c	*P*2₁/*c*
a/Å	12.6091(7)	5.56720(10)	7.54590(10)	12.1442(5)
b/Å	6.5090(5)	17.1985(4)	13.5083(2)	6.9640(3)
c/Å	9.9212(5)	10.0040(2)	19.3375(3)	14.1111(6)
α/°	90	90	90	90
β/°	90	93.848(2)	93.3310(10)	93.260(4)
γ/°	90	90	90	90
Volume/Å^3	814.26(9)	955.70(3)	1967.79(5)	1191.48(9)
Z	4	4	4	4
ρ_{calc} /g cm^{-3}	1.441	1.585	1.850	1.418
μ/mm^{-1}	0.650	3.304	13.098	3.874
F(000)	368.0	464.0	1088.0	536.0
Radiation	Mo Kα (λ = 0.71073)	Cu Kα (λ = 1.54184 Å)	Cu Kα (λ = 1.54184)	Cu Kα (λ = 1.54184)
2Θ range for data collection/°	8.156 to 52.998	10.246 to 132.79	7.988 to 132.996	7.292 to 133.898
Index ranges	$-15 \le h \le 15$, $-5 \le k \le 8$, $-11 \le l \le 12$	$-6 \le h \le 5$, $-20 \le k \le 20$, $-11 \le l \le 11$	$-8 \le h \le 8$, $-16 \le k \le 16$, $-22 \le l \le 20$	$-14 \le h \le 14$, $-5 \le k \le 8$, $-16 \le l \le 16$
Reflections collected	3050	10548	15351	8232
Independent reflections	914 [R_{int} = 0.0371, R_{sigma} = 0.0350]	1689 [R_{int} = 0.0595, R_{sigma} = 0.0374]	3450 [R_{int} = 0.0343, R_{sigma} = 0.0281]	2117 [R_{int} = 0.0305, R_{sigma} = 0.0260]
Data/restraints/parameters	914/0/62	1689/256/174	3450/0/237	2117/0/148

Goodness-of-fit on F^2	1.083	1.064	1.084	1.076
Final R indexes [I>=2σ(I)]	R_1 = 0.0333, wR_2 = 0.0824	R_1 = 0.0777, wR_2 = 0.1856	R_1 = 0.0247, wR_2 = 0.0641	R_1 = 0.0298, wR_2 = 0.0803
Final R indexes [all data]	R_1 = 0.0391, wR_2 = 0.0868	R_1 = 0.0798, wR_2 = 0.1872	R_1 = 0.0262, wR_2 = 0.0649	R_1 = 0.0321, wR_2 = 0.0823
Largest diff. peak/hole / e Å^{-3}	0.38/−0.32	0.53/−0.42	0.56/−0.71	0.23/−0.25
CCDC	2105605	2105606	2105607	2105608
